# Development of CAR-T Cell Persistence in Adoptive Immunotherapy of Solid Tumors

**DOI:** 10.3389/fonc.2020.574860

**Published:** 2021-01-06

**Authors:** Jiaqiao Fan, Jugal Kishore Das, Xiaofang Xiong, Hailong Chen, Jianxun Song

**Affiliations:** ^1^Department of General Surgery, The First Affiliated Hospital of Dalian Medical University, Dalian, China; ^2^Department of Microbial Pathogenesis and Immunology, Texas A&M University Health Science Center, Bryan, TX, United States

**Keywords:** chimeric antigen receptor, T cells, persistence, solid tumor, immunotherapy

## Abstract

Chimeric antigen receptor (CAR) T (CAR-T) cell transfer has made great success in hematological malignancies, but only shown a limited effect on solid tumors. One of the major hurdles is the poor persistence of infused cells derived from *ex vivo* activation/expansion and repeated antigen encounter after re-infusion. Bcl-xL has been demonstrated to play an important role on normal T cell survival and function as well as genetically engineered cells. In the current study, we developed a retroviral CAR construct containing a second-generation carcinoembryonic antigen (CEA)-targeting CAR with the Bcl-xL gene and tested the anti-CEA CAR-T cell immunotherapy for colorectal cancer. *In vitro*, the anti-CEA CAR-T cells destroyed CEA-expressing tumor cells and sustained survival. *In vivo*, adoptive cell transfer of anti-CEA CAR-T cells significantly enhanced the ability of the CAR-T cells to accumulate in tumor tissues, suppress tumor growth and increase the overall survival rate of tumor-bearing mice in a murine model of colorectal cancer. These results demonstrate a novel CAR-T platform that has the ability to increase the persistence of CAR-T cells in solid tumors through exogenous expression of persistent genes. The data provide a potentially novel approach to augment CAR-T immunotherapy for solid tumors.

## Introduction

Immunotherapy especially immune checkpoint inhibitors (ICI) and CAR-T therapy is emerging as one of the most promising approaches for relapse and refractory malignancies in the past decades (1). Only a fraction of patients with solid tumors benefit from ICI with high relapse rates and adverse event rate (2). For CAR-T therapy, although a great progress has been made in hematological malignancies (3), but numerous challenges remain unsolved in solid tumors (4). For instance, selecting favorable target antigens to avoid on-target off-tumor adverse effect; enhancing the ability of infused cells to accumulate in the high-density tumor lesion; and improvement of proliferation and/or persistence of the infused cells within the tumor mass (5). Amongst these obstacles, the lack of proliferation and/or persistence may be responsible to a great extent for the poor effect of CAR-T therapy in solid tumors. Moreover, even for hematological malignancies with complete remission rate as high as to 90%, the patients remain at the risk of relapse because of the poor persistence of CAR-T cells *in vivo*. Effective strategies to circumventing these barriers should significantly improve current tumor immunotherapy, thus are urgently needed (6).

Various factors could influence the persistence of CAR-T cells, including patient preconditioning procedure (7), *ex vivo* culture conditions (8), and the molecular design of CARs (9, 10). In addition to improving the patient precondition approaches and *ex vivo* cell culture protocols, optimization of the CAR molecule structural design is the most common strategies to increase the CAR-T function and persistence. It is well established that the costimulation receptor is critical for T cell activation and proliferation, and that modification of CAR molecules with variable costimulation domains is one of the preferred methods to enhance the persistence of CAR-T cells (11, 12). In fact, almost all CARs possess one or more costimulation domains designated the second or third generation of CARs respectively. More recently, the fourth generation of CARs containing inducible transgenes (termed TRUCKs) and capable of constantly secreting cytokines (e.g., IL-12, IL-15, or IL-18) (13–15) has been subsequently tested. The extracellular non-signal structure such as spacers and transmembrane domains could also be modified to improve the persistence of CAR-T cells *in vivo* (16, 17). Even though some progress has been made, antitumor T cell persistence remains the major hurdle of CAR-T therapy for solid tumors in preclinical and clinical research.

The comprehensive mechanism of CAR-T cell persistence remains to be identified. Activation induced cell death (AICD) may play an important role since the therapeutic T cells encounter stimulation all the way from activation, proliferation/expansion, genetically edition to infusion. The immune suppressive microenvironment may be another important mechanism to inhibit the persistence of CAR-T cells in tumor lesion. A number of suppressive regulatory cells and cytokines could either keep CAR-T cells from tumor tissues or induce CAR-T cell apoptosis in site (5). In addition, the tumor microenviroment lacking various cytokines necessary for T cell persistence can accelerate the apoptotic process. However, almost all the methodologies mentioned above could resolve only part of these problems. Nevertheless, enhancing the ability of T cell persistence directly among the entire process of CAR-T production and treatment may be a more favorable approach.

B-cell lymphoma-extra large (Bcl-xL) plays an important role in T cell survival and function (18, 19), especially after activation. We and other group have previously reported that Bcl-xL overexpression could substantially improve the persistence and antitumor ability of antigen-specific T cells (20, 21), another group demonstrated that Bcl-xL expression resulted from costimulation signal contributes to CAR-T persistence and tumor eradication (22). We hypothesized that increasing Bcl-xL expression is a feasible strategy to enhance the ability of CAR-T cell resistant to AICD and the suppressive tumor microenvironment, therefore improving their persistence and antitumor reactivity. In the present study, we included an exogenous Bcl-xL gene into a second-generation anti-CEA CAR retroviral construct. After gene transduction of T cells, we observed a high expression of exogenous Bcl-xL in the transduced cells, and the CAR-T cells containing exogenous Bcl-xL gene promoted its persistence both *in vitro* and *in vivo*. Importantly, adoptive cell transfer of the anti-CEA CAR-T cells in a mouse model of colorectal cancer significantly suppressed tumor growth and sustained mouse survival, suggesting the anti-CEA CAR-T cells had potent antitumor ability. Our strategy of the CAR design used in this experiment may be explored as a new platform to overcome the main obstacle for CAR-T therapy in solid tumors as well as hematological malignancies.

## Materials and Methods

### Cell Lines and Mouse

MC-38 cell line is CEA negative murine colon cancer cell derived from C57BL/6 mice, and MC-38-CEA-2 (MC-32) cells are CEA positive MC-38 cells retrvirally transduced with human CEA gene (23). Both cell lines were obtained from Kerafast Inc. (Boston, MA). C57BL/6 (B6) and Thy1.1 congenic mice (B6.PL-*Thy1a*/CyJ) were purchased from The Jackson Laboratory (Bar Harbor, ME, USA).

### Antibodies

β-actin (#4967), Bcl-xL (#2762), Myc (#14819 and #12855) and peroxidase-conjugated anti-rabbit (#7054) or anti-mouse Ig (#7056) for Western blot, were obtained from Cell Signaling Technology (Beverly, MA). Anti-mouse CD3 (145-2C11) and CD28 (37.51) antibodies, mouse IL-2 and IFN-γ ELISA Kits, all FITC-, PE-, PE/Cy5-, PE/Cy7, and APC-conjugated antibodies, PE Annexin V Apoptosis Detection Kit (640934) and Cyto-Fast™ Fix/Perm Buffer Set (#426803) were purchased from Biolegend (San Diego, CA)

### Cell Cultures

Tumor cells were thawed in 37°C and washed with fresh RPMI-1640 media twice before incubation, and cultured with 5% CO_2_ at 37°C in complete RPMI-1640 media supplemented with 10% heat-inactivated FBS, 1% penicillin/streptomycin and 1% L-glutamine. Retrovirus-packaging Plat-E cells expressing retroviral particles pol, gag and env to package the retroviral vector DNA coding sequences to produce retroviruses without the probability of generating replication competent viruses (24) were used for retroviral transduction. The expansion of Plat-E cells began on day 4 before the retrovirus packaging operation and were passaged at the density of 3×10^6^ cells in 10 ml of DMEM per 10cm plate at least 16 h before transfection. Naive CD8^+^ T cells were purified from the spleen and lymph nodes of C57BL/6 mice by using the murine naive CD8a^+^ T cell isolation kit (#130-096-543, Miltenyi Biotech, CA) and cultured in complete media additionally supplemented with 0.06% β-mercaptoethanol based on the complete RPMI-1640 media.

### Retroviral Transduction

Anti-human CEA (hMN14) (CD28-CD3ζ) CAR and cDNA for Bcl-xL was linked with 2A sequence and subcloned into the retroviral vector MFG (20, 25), which was derived from murine leukemia virus (MLV)-based retroviral vector and had been demonstrated ready to be modified to improve safety and gene expression (26). Three constructs used in the present study are Vector 1: MFG-control vector (empty plasmid without interest gene); Vector 2: MFG-anti-CEA CAR (MFG-based vector subcloned an anti-CEA CAR sequence); and Vector 3: MFG-anti-CEA CAR+Bcl-xL (MFG-based vector with the same CAR with Bcl-xL gene). The CAR structure in the MFG constructs consists of a synthetic single chain variable fragment (scFv, derived from mouse monoclonal antibody, clone MN-14) specific to hCEA epitope expressed on MC38-CEA-2 cells in which a Myc epitope-tagged framework was engineered to enable the tracking of scFV expression, a spacer and a transmembrane domain (both derived from CD28), a single costimulation domain (derived from human CD28), and a T cell receptor (TCR) signaling element (derived from human CD3ζ chain). CAR in Vector 3 contains a Bcl-xL gene with CD3 ζ through 2A element. The parental MFG-based retroviral plasmids were expanded in MAX Efficiency™ DH5α Competent Cells (ThermoFisher, #18258012) and purified with Invitrogen™ PureLink™ Expi Endotoxin-Free Maxi Plasmid Purification Kit (ThermoFisher, #A31231) according to the manufacturer’s instruction. All constructs were verified by sequencing.

Retroviral transduction was performed as described before (27). Briefly, Plat-E packaging cells using the ecotropic envelope were transfected with the CAR constructs and supernatants from the transfected packaging cells were collected 48 h after transfection, and used for transduction of T cells. Murine mature T cells from lymph nodes and spleen were used in all experiments. 5 ×10^5^ CD8^+^ T cells were stimulated with anti-CD3 plus anti-CD28 antibodies. After 2 days, the supernatant was replaced with 1 ml viral supernatant containing 5 µg/ml Polybrene (Sigma), and the cells were spun for 1 h at 32°C and incubated at 32°C for 8 h. This was repeated the following day. Viral supernatant was removed and replaced with fresh medium, and T cells were re-cultured. Expression of GFP was determined by flow cytometry gating on CD8^+^ T cells. GFP-expressing T cells were purified from cell sorting using a FACS Vantage SE I high-speed cell sorter (BD Immunocytometry Systems, San Jose, CA).

### Cytokine Secretion, Cell Recovery, and Proliferation

5 ×10^5^ CD8^+^ T cells were stimulated with anti-CD3 plus anti-CD28 antibodies in 48-well plates. Cytokines were measured by enzyme-linked immunosorbent assay (ELISA; Biolegend); T cell survival *in vitro* was determined by trypan blue (Sigma) exclusion assay; and proliferation was measured in triplicate cultures by incorporation of ^3^H-thymidine (1 µCi/well; ICN Pharmaceuticals, Laval, QC, Canada) during the last 12 h of culture (20).

### Immunoblotting

Live CD8^+^ cells were recovered by Ficoll treatment and positive selection with anti-CD8 microbeads (Miltenyi Biotec Inc). Cells lysates were extracted and used for Western blotting as described (28).

### *In Vitro* Cytotoxicity Assay

Target MC-38 (hCEA^-^) or MC-32 (hCEA^+^) tumor cells were co-cultured with effector CEA CAR-T cells at the target:effector (E:T) ratio in 1:2, 1:5, or 1:10 in triplicate. For test of background, wells contained target cells only. The plates were incubated at 37**°**C for 12 h, and the CAR-T cell-mediated cytotoxicity was measured using the Cayman’s 7-AAD/CFSE Cell-Mediated Cytotoxicity Assay Kit according to the manufacture’s instruction by flow cytometry. In brief, both prepared MC-38 and MC-32 cells were stained with CFSE dye prior to co-culture with CAR-T cells according to the protocols previously described (29). The percentage of specific lysis was calculated as follows: cytotoxicity (%): [100% × dead targets/(dead targets + live targets)] (experiment) *−* [100% × dead targets/(dead targets + live targets)] (background). In addition, an Amnis ISXII (MilliporeSigma) equipped with 405, 488, and 642 nm lasers with a single camera (six channels) was used to acquire experimental samples using the INSPIRE software.

### Adoptive Transfer and Tumor Challenge

T cells were cultured with anti-CD3 plus anti-CD28 antibodies and transduced on day 2 and 3 with retroviral vectors (27). Cells were re-cultured for 2 more days. GFP^+^ CD8^+^ T cells were sorted and 3 × 10^6^ sorted cells were injected *i.v.* into C57BL/6 mice, which were challenged *s.c.* with 5 × 10^6^ MC-38 or MC-32 tumor cells in PBS, or PBS without tumor cells as a control one week prior to the adoptive T cell transfer. The volume of the tumor (mm^3^) was measured using a caliper by a blinded investigator and calculated as follows: V = length × width^2^ × 0.52. Mice were sacrificed when the tumor size reached 20mm in any direction.

### Histology and Immunofluorescence

Tumor tissues were collected from mice at the indicated time points. Samples were collected in embedding cassette and blocked with 10% neutral buffered formalin. Samples were infiltrated with the wax and embedded the infiltrated tissues into wax blocks. Both vertical and horizontal sectioning were prepared for immunostaining.

H&E staining: Routine Hematoxylin & Eosin (H&E) staining was performed at an interval of every five serial sections. Immunological staining: Tissue sections were fixed with acetone (Sigma), and incubated with 3% bovine serum albumin (BSA; Sigma) to block non-specific protein binding (30). Sections were stained with fluorescein isothiocyanate (FITC) anti-Myc (1:1000; Thermo Fisher #13-2511).

### Statistical Analysis

The data were presented as the mean ± standard error of mean (SEM). Differences in means were analyzed by Student’s t-test. One-way ANOVA with Tukey *post hoc* tests were used for differences between three or more groups in a single condition or time point. Survival curves were constructed by the Kaplan−Meier method and analyzed with the log-rank test. All tests were 2-sided, with *p* < 0.05 considered to indicate statistical significance. All statistics were calculated using GraphPad Prism 8 (San Diego, CA, USA).

## Results

### Expression of Anti-Carcinoembryonic Antigen Chimeric Antigen Receptor and Bcl-xL in Primary CD8+ T Cells

**Figure 1:**
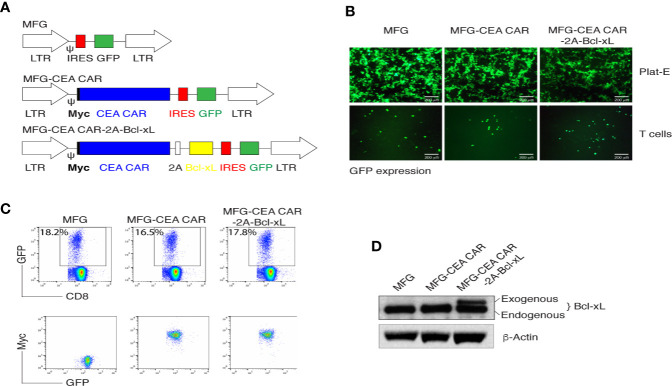
*In vitro* generation of carcinoembryonic antigen (CEA) chimeric antigen receptor T (CAR-T) cells. **(A)** Schematic representation of the retroviral constructs MFG, MFG-CEA CAR, and MFG-CEA CAR-2A-Bcl-xL. CEA CAR is Myc tagged; Ψ, packaging signal; 2A, picornavirus self-cleaving 2A sequence; LTR, Long terminal repeats. **(B)** The packaging Plat-E cells (upper panel) and MFG-transduced CD8^+^ T cells (lower panel) were visualized by fluorescence microscopy (scale bars: 200 μm). **(C)** CD8^+^ T cells were transduced with the retroviral constructs and GFP^+^CD8^+^ T cells (upper panel) or GFP^+^Myc^+^ T cells gated on GFP^+^CD8^+^ populations were analyzed by flow cytometry. **(D)** GFP^+^CD8^+^ T cells were sorted, and the cell lysates were determined for the expression of Bcl-xL and β-actin by western blotting. All data are representative of three independent experiments.

MFG-based retroviral vector had been demonstrated a reliable gene-edition system with high transduction efficiency and enable engineered cells highly expressing the interest protein (31). The 2A peptide regions from Picornavirus has been widely used to create an individual fragment encoding multiple proteins (32). To generate reliable and versatile constructs to transduce primary CD8^+^ T cells that permit the expression of multiple genes, we used a T2A sequence to generate multicistronic retroviral vectors with efficient translation of two cistrons (e.g., the CEA scFv-TM28-CD28-CD3ζ CAR with Myc tag and Bcl-xL) (**Figure 1A**). The CEA scFv was derived from mouse monoclonal antibody clone MN14.

Thus, two fragments of the CEA CAR and Bcl-xL were linked with the 2A sequence and were subcloned into the MFG vector. The new construct MFG-CEA CAR-2A-Bcl-xL was confirmed by DNA sequencing (**Supplementary Data 1**) and GFP expression in the packaging Plat-E and primary T cells (**Figure 1B**). Furthermore, naive CD8^+^ T cells were infected with the retrovirus-mediated transduction, which led to the surface expression of CEA CAR (Myc^+^) (**Figure 1C**) and exogenous expression of Bcl-xL (32 kDa; **Figure 1D**).

### Bcl-xL Promoted the Survival of Carcinoembryonic Antigen Chimeric Antigen Receptor-T Cells *In Vitro*

**Figure 2:**
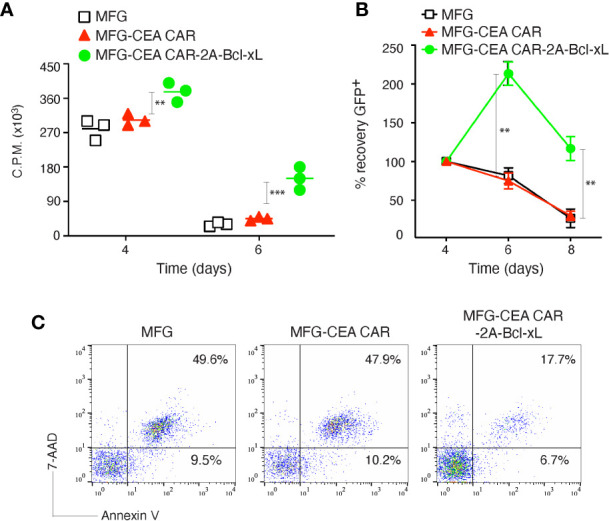
Carcinoembryonic antigen (CEA) chimeric antigen receptor T (CAR-T) cells overexpressing Bcl-xL had enhanced proliferation and survival *in vitro*. Naive CD8^+^ T cells from C57BL/6 mice were stimulated with anti-CD3 plus anti-CD28 antibodies, and transduced on days 2/3 with retroviral vectors expressing GFP, GFP with CEA CAR, or GFP with CEA CAR and Bcl-xL, and then re-cultured without any further stimulation. **(A)** Primary passive proliferation on day 4 and day 6 were measured in unseparated cultures by pulsing with tritiated thymidine for 20 h. Data are represented with a mean of three independent experiments (**P < 0.01, ***P < 0.001, Student’s unpaired *t*-test). **(B)** GFP^+^CD8^+^ T cell recovery normalized to take into account differences in initial transduction efficiency between cultures. Numbers of GFP^+^ cells present on day 4 were assigned a value of 100%, and numbers surviving on day 6 and day 8 were used to calculate the percentage recovery relative to day 4. The data represent the mean ± S.E.M. percentage change from three separate experiments (*P < 0.05, **P < 0.01, Student’s unpaired *t*-test). **(C)** Apoptosis of GFP^+^ CD8^+^ T cells on day 6 based on staining of Annexin V and 7-AAD and analyzed by flow cytometry. Data are representative of three independent experiments.

To determine whether enforced expression of Bcl-xL could contribute to the survival of CEA CAR-T cells, we compared the proliferation, recovery and apoptosis rate of transduced CD8^+^ T cells with the MFG retroviral constructs containing Bcl-xL or not. The primary CD8^+^ T cells were activated with anti-CD3 plus CD28 antibodies, and after retroviral transduction on days 2-3, T cells were passively re-cultured in the absence of further stimulation and their proliferation was assessed by thymidine incorporation after 1 (day 4) and 3 days (day 6). Bcl-xL-forced expression in CD8^+^ T cells resulted in enhanced passive proliferation at late times at day 6, as measured by thymidine incorporation, compared to only CEA CAR expression (**Figure 2A**). In line with this, enumerating the recovery of live T cells through monitoring GFP expression showed that expression of Bcl-xL allowed CD8^+^ T cells to expand from day 4 through day 6 over that engendered by transducing only CEA CAR in isolation. Culture over 8 days showed that, Bcl-xL significantly enhanced the ability of CD8^+^ T cells to survive (**Figure 2B**). In addition, the percentage of apoptosis in CD8^+^GFP^+^ T cells was reduced in anti-CEA CAR+Bcl-xL transduction group compared to transfection of only anti-CEA CAR (**Figure 2C**).

**Figure 3:**
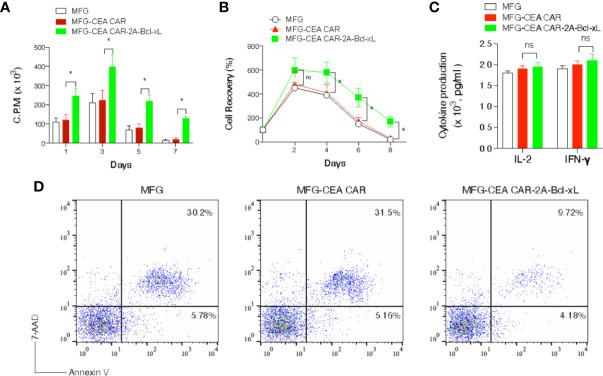
Carcinoembryonic antigen (CEA) chimeric antigen receptor T (CAR-T) cells overexpressing Bcl-xL had a greater recall response *in vitro*. GFP^+^CD8^+^ T cells were sorted (**Figure 2**) and re-stimulated with anti-CD3 plus anti-CD28 antibodies. **(A)** Recall proliferation on various days was measured in by pulsing with tritiated thymidine for 20 h. Data are representative with mean ± S.E.M. percentage change from three independent experiments (*P < 0.05, Student’s unpaired *t*-test). **(B)** Recall survival on various days. The data represent the mean ± S.E.M. percentage change from three independent experiments (*P < 0.05; ns, not significant, Student’s unpaired *t*-test). **(C)** Recall IL-2 and IFN-γ production by ELISA at 40 h. ns, not significant. Data are means ± S.E.M. from three experiments. **(D)** Apoptosis of GFP^+^ CD8^+^ T cells on day 6 based on staining of Annexin V and 7-AAD and analyzed by flow cytometry. Data are representative of three independent experiments.

To investigate whether overexpression of Bcl-xL promote greater recall responses of CEA CAR-T cells, effector CD8^+^ T cells from C57BL/6 mice expressing CEA CAR or CEA CAR with Bcl-xL from primary naive cultures were sorted based on GFP expression and re-stimulated. CD8^+^ T cells transduced with CEA CAR with Bcl-xL displayed enhanced recall proliferation (**Figure 3A**), and greater numbers were recovered over time (**Figure 3B**) compared to the introduction of CEA CAR alone in isolation. In addition, effector function was not affected in which production of IL-2 or IFN-γ was unaltered regardless of the forced expression of Bcl-xL (**Figure 3C**). However, the percentage of apoptosis in CD8^+^GFP^+^ T cells transduced with CEA CAR with Bcl-xL was decreased compared to the introduction of CEA CAR alone in isolation. The percentage of early apoptotic cells (Annexin V^+^7-AAD^-^) reduced less (4.18% with CEA CAR with Bcl-xL *vs.* 5.78% in MFG vector control and 5.16% with CEA CAR alone), but the percentage of late apoptotic cells (Annexin V^+^ 7-AAD^+^) markedly lessened (9.72% with CEA CAR with Bcl-xL *vs.* 30.2% in vector control and 31.5% with CEA CAR alone) (**Figure 3D**).

Collectively, our results demonstrated that expression of Bcl-xL substantially promotes the survival of CEA CAR-T cells when measuring short-term T cell expansion *in vitro*.

### The Cytotoxicity of Carcinoembryonic Antigen Chimeric Antigen Receptor-T Cells Was Unaffected by Overexpression of Bcl-xL *In Vitro*

**Figure 4:**
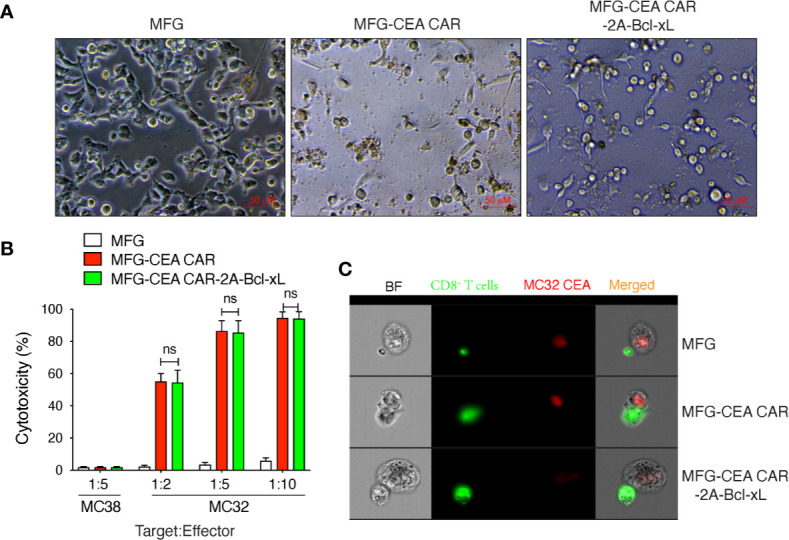
Carcinoembryonic antigen (CEA) chimeric antigen receptor T (CAR-T) cells overexpressing Bcl-xL had modest *in vitro* cytotoxicity. GFP^+^CD8^+^ T cells were sorted (**Figure 2**), and co-cultured with MC-38 or MC-32 tumor cells *in vitro*. The *in vitro* cytotoxicity was measured by the Cayman’s 7-AAD/CFSE Cell-Mediated Cytotoxicity Assay Kit. **(A)** Representatives of the *in vitro* co-culture of T cells with MC-32 tumor cells at 12 h (scale bars: 50 μm). **(B)** *In vitro* cytotoxicity assay. Data are representative with mean ± S.E.M. from three independent experiments (ns, not significant, *P*>0.05, Student’s unpaired *t*-test). **(C)** Representatives of the *in vitro* colocalization of CAR-T cells and tumor cells by Amnis image stream analysis. Data are representative of three independent experiments.

To determine this functional activity of CEA CAR-T cells, we co-cultured the CAR-T cells with murine tumor cells: MC-32 (expressing human CEA) and MC-38 (lacking human CEA) (**Figure 4A**). No apparent cytotoxicity was observed in CEA negative MC-38 cells at the target:effect or ratio of 1:5 for each transduced cell. The control T cells showed no cytotoxicity to MC-32 cells at any target:effector ratio and the CEA CAR-T cells showed significantly increased cytotoxicity to MC-32 cells in the co-culture system at various target:effector ratio of 1:2, 1:5, 1:10 (**Figure 4B**), indicating a CEA-specific antitumor reaction. Furthermore, the CAR-T cells over-expressing Bcl-xL or not displayed a dosage-dependent cytotoxicity to MC-32 cells, but no significant difference was observed between these two groups, indicating that the exogenous Bcl-xL protein did not affect on the overall antitumor function of the CAR-T cells *in vitro*.

Amnis image stream analysis showed colocalization of CAR-T cells and tumor cells, which suggest that the scFv of the CAR expressed on CEA CAR-T could bind with hCEA on tumor cell stably and activated the CAR-T sufficiently, because both CAR-T with or without Bcl-xL were far bigger than T cell transduced with empty MFG vector. The data also indicate that the cytotoxicity of CAR-T is cell-cell direct-contact dependent (**Figure 4C**).

### Bcl-xL Promoted the Persistence of Carcinoembryonic Antigen Chimeric Antigen Receptor-T Cells *In Vivo*

**Figure 5:**
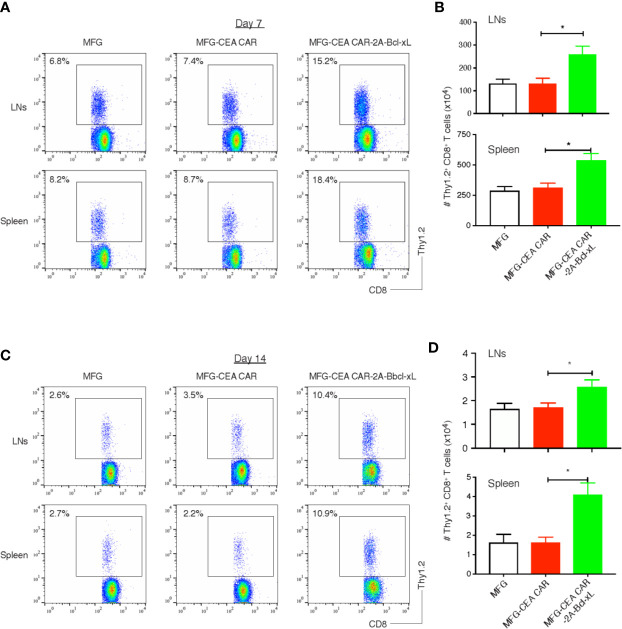
Bcl-xL promoted the persistence of carcinoembryonic antigen (CEA) chimeric antigen receptor T (CAR-T) cells *in vivo*. 3×10^6^ GFP^+^ CD8^+^ T cells (Thy1.2^+^, **Figure 2**) were adoptively transferred into Thy1.1 congenic mice *via* the tail vein, and on day 7 and day 14, CD8^+^ Thy1.2^+^ T cells in the lymph nodes (LNs) and spleen were analyzed by flow cytometry. **(A)** Representatives of the CD8^+^ Thy1.2^+^ T cells in the LNs and spleen on day 7. Data are representative of three independent experiments. **(B)** Cell numbers of CD8^+^ Thy1.2^+^ T cells in the lymph nodes (LNs) and spleen on day 7. Data are representative with mean ± S.E.M. percentage change from three independent experiments (*P<0.05, Student’s unpaired *t*-test). **(C)** Representatives of the CD8^+^ Thy1.2^+^ T cells in the LNs and spleen on day 14. Data are representative of three independent experiments. **(D)** Cell numbers of CD8^+^ Thy1.2^+^ T cells in the LNs and spleen on day 14. Data are representative with mean ± S.E.M. percentage change from three independent experiments (*P < 0.05, Student’s unpaired *t*-test).

To determine whether Bcl-xL was capable of increasing the expansion or persistence of CEA CAR-T cells in response to antigen presented *in vivo*, GFP-sorted CEA CAR-T cells (Thy1.2^+^), obtained from the *in vitro* cultures in **Figure 3** and adoptively transferred into syngeneic recipients (Thy1.1^+^). These mice were subsequently challenged with anti-CD3 CD3ϵ F(ab’)_2_ fragment. Activated T cells transduced with either the vector control or the CEA CAR expanded less over 3 days in the lymph nodes and spleen than those CEA CAR-T cells expressing Bcl-xL (**Figures 5A, B**), supporting the *in vitro* results (**Figure 3**). The effect of Bcl-xL is long lasting, with enhanced numbers of CEA CAR-T cells not only present a week after the anti-CD3 challenge through the peak of response, but also after two weeks while the secondary *in vivo* response was completed and contraction of T cell populations had followed in all recipients (**Figures 5C, D**). Overall, these data strongly support the conclusion that an action of Bcl-xL sustains the persistence of CEA CAR-T cells.

### Adoptive Cell Transfer of Carcinoembryonic Antigen Chimeric Antigen Receptor-T Cells Overexpressing Bcl-xL Significantly Increased T Cell Accumulation Within Tumor Tissues and Suppressed Tumor Growth

**Figure 6:**
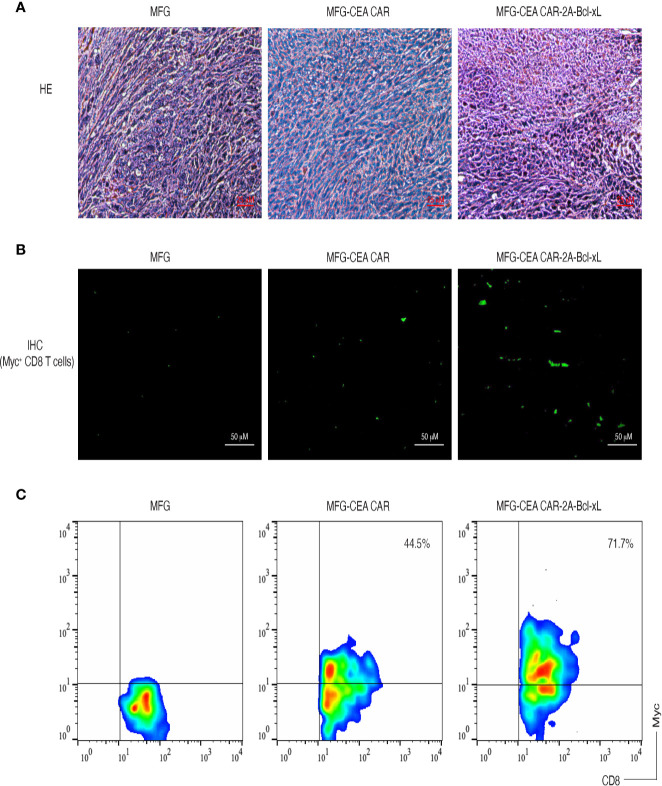
Carcinoembryonic antigen (CEA) chimeric antigen receptor T (CAR-T) cells overexpressing Bcl-xL prominently accumulated in tumor tissues. C57BL/6 mice were adoptively transferred with CEA CAR-T cells expressing with or without Bcl-xL or control cells one week after the mice were *s.c.* injected in the flank region with MC-38 or MC-32 tumor cells. On day 21 to 22 after tumor challenge, tumor tissues were examined for tumor-reactive T cell infiltration. **(A)** H&E staining (scale bars: 25 μm). **(B)** Immunohistological staining (scale bars: 50 μm). Myc^+^ CEA CAR-T cells (green) infiltrated in tumor tissues (the dark background). **(C)** Single-cell suspensions from tumor tissues were analyzed for expression of CD8^+^ and Myc^+^ T cells by flow cytometry, after gating on the CD8^+^ population. Data are representative of three independent experiments.

**Figure 7:**
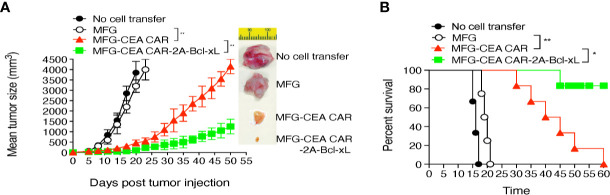
Adoptive cell transfer of carcinoembryonic antigen (CEA) chimeric antigen receptor T (CAR-T) cells expressing Bcl-xL substantially suppressed tumor growth and sustained mouse survival. The tumor challenge and the adoptive cell transfer are described in **Figure 6**. **(A)** Tumor growth was monitored over time with representative tumors on day 18. Data are mean tumor size ± S.D. from six individual mice (n=6) and representative of three experiments. **(B)** Mouse survival was assessed over 60 days (Kaplan–Meier survival analysis). *P < 0.05, **P < 0.01, Student’s unpaired t-test.

To demonstrate that the CEA CAR-T cells overexpressing Bcl-xL have the ability in inducing CEA-specific T cell persistence in a physiologically and clinically relevant setting, we used a murine model of colorectal cancer (**Supplementary Data 2**). We *s.c.* injected C57BL/6 mice in the flank region with MC-32 or control MC-38 tumor cells. After a week, we performed *i.v.* adoptive cell transfer of genetically engineered T cells. Three weeks after the adoptive cell transfer, we observed that more CEA-reactive CD8^+^ T cells accumulated in MC-32 tumor tissues in mice receiving CEA CAR-T cells overexpressing Bcl-xL than those receiving the CEA CAR-T cells without overexpressing Bcl-xL or the non-specific T cell control (**Figure 6**). Mice receiving CEA CAR-T cells overexpressing Bcl-xL had the smaller MC-32 tumor sizes as compared to those receiving the CEA CAR-T cells without overexpressing Bcl-xL or the non-specific T cell control (**Figure 7A**), correlating with their enhanced survival (n=6; **Figure 7B**). Taken together, these findings indicate that the adoptive transfer of CEA CAR-T cells overexpressing Bcl-xL can generate CEA-specific CTL persistence and result in enhanced tumor suppression.

## Discussion

CAR-T cell persistence has been associated with better clinical outcome in both patients with blood malignancies and solid tumors (33, 34). The programmed cell death through various mechanisms may be responsible for CAR-T cell apoptosis and short-term persistence (35). Numerous strategies have been tested by different groups and obtained promising results (11–16), however, an optimized condition remains to be identified. We and other group have previously reported that both exogenous and endogenous Bcl-xL could improve the persistence and antitumor ability of antigen-specific T cells (20, 21). In the current study, we exploited in a novel approach to enhance the ability of CAR-T cell persistence by overexpressing exogenous Bcl-xL and demonstrated promising results. The constructs used in the study could be an excellent platform for CAR design to increase CAR-T cell persistence in solid tumors.

Endogenous Bcl-xL is encoded by a Bcl-2 gene superfamily, expresses on mitochondrial membrane and binds specifically with the residues of cytochrome C, the process could prevent cytochrome C released from mitochondria to initiate the programmed cell death (36). It was demonstrated that Bcl-xL can rescue T cells from TCR engagement mediated cell death (37). Bcl-xL is the critical driver of T cell expansion, not only through enhancing activated T cell survival, but also increasing T cell proliferation and energy metabolism (38). Bcl-xL could also rescue T cells from PD-1 and/or Fas signal-induced cell death (18, 39), which generally upregulate at the early stage of T cell activation. Several costimulation signals were demonstrated to involve in upregulation of the expression of endogenous Bcl-xL in activated T cells (40–43), further indicating the important role of Bcl-xL in the life cycle of T cells. Therefore, various strategies combining distinct costimulatory domains with the CAR gene have been tried to increase Bcl-xL expression in CAR-T cells and achieved promising results (44). Since Bcl-xL provides an intrinsic mechanism for activating T cell survival, we introduce the Bcl-xL gene with a second-generation CAR through the 2A sequence, then exogenous Bcl-xL can be transcribed along with the CAR. We confirmed high expression of exogenous Bcl-xL protein in MFG-anti-CEA CAR+Bcl-xL transduced T cells and no exogenous Bcl-xL (~32 kDa) is expressed in MFG-anti-CEA CAR transduced T cells as well as empty MFG vector and untransduced T cells. The expression level of endogenous Bcl-xL is comparable amongst variable transduced and untransduced T cells, indicating exogenous Bcl-xL gene incorporating has no effect on the expression of endogenous Bcl-xL. In *ex vivo* experiment, both CEA specific CAR-T cells with or without exogenous Bcl-xL gene displayed significant cytotoxicity than the empty control vector. Bcl-xL did not improve cytotoxicity of the CAR-T cells *in vitro*; however, the *in vivo* study clearly showed that CAR-T cells expressing Bcl-xL considerably enhanced their antitumor ability.

*In vivo* persistence of CAR-T cells was notably enhanced by exogenous expression of Bcl-xL. IHC images showed increased accumulation of CAR-T cells expressing Bcl-xL, and flow cytometry analysis presented that the frequency of MFG-anti-CEA CAR+Bcl-xL transduced T cells was significantly higher than that of MFG-anti-CEA CAR transduced T cells in tumor-infiltrating lymphocytes (TILs), further indicating improved persistence of CAR-T cells by overexpressed exogenous Bcl-xL. These results may interpret the enhanced antitumor potency of CAR-T cells expressing Bcl-xL. Further analysis comparing the frequency of CAR-T cells in various tissues demonstrated the accumulation of CEA-specific CAR-T cells in the tumor lesion but not in the lymph nodes and spleen, indicating that Bcl-xL-induced CAR-T cell accumulation is antigen-specific.

Targeting antigen selection is another critical issue for CAR-T therapy because almost all of the tumor-associated antigens are shared by normal tissue/organs at a relatively low level. On-target off-tumor effect is one of the major obstacles for CAR-T therapy in solid tumors, and generally results in lethal adverse effect once the antigen expresses on life-important organ even at a very low level (45). The total number of available targeting antigens for CAR-T treatment in clinical trials for solid tumors is very limited, considering the amount of tumor types, it is very few of each type of tumor (46). CEA is considered as a relatively safe and effective target, it is expressed at high levels in embryo phase and decrease rapidly to very low level after birth, and majorly expressed at colorectal mucus apical side. CEA is greatly overexpressed in most epithelial cell-derived cancers (47), and often used as a representative tumor-associated antigen to investigate immunotherapy for solid tumors. In the current study, we demonstrated that CEA is an effective target for CEA-specific CAR-T cells and effectively recruit CAR-T cells into the tumor lesion. Consideration of the CAR designation platform containing human T cell activation signal as well as hCEA targeting scFv, it is ready to test the efficiency of the constructs engineered human T cells against various human solid tumors expressing CEA through *in vitro* system, such as co-culture with organoid as previously described (48). Another advantage of the constructs used in the study is the MFG-based vector, which is the retroviral vector used extensively in clinical trials and ready to be modified by inserting IRES and multicloning sites without adverse effects (26).

The phenotype of persistent CAR-T cells remains to be identified. It had been demonstrated that adoptive transfer of genetically edited memory T cells has long-term persistence (49), and Bcl-xL contribute to sustain effector and memory T cells at later stages of activation through members of the TNFR family (50, 51). We acknowledge the potential oncogenic risk of exogenous Bcl-xL expression. Apoptosis is the general mechanism to eliminate cells with damaged DNA or aberrant cell cycle, anti-apoptotic proteins have therefore oncogenic potential. Although mature T cells seem resistance to gene transfer even for oncogene (52), and we examined that most anti-CEA CAR-T cells did not survive after a month of the adoptive cell transfer, a longer observation of CAR-T cells expressing exogenous Bcl-xL is needed prior to a further clinical trial. As an alternative, the depletion of the transferred CAR-T cells can be achieved by targeting the inserting suicide genes into the CAR construct, or designation of a universal target for accessible defined therapeutic monoclonal antibody in the external domain of the CAR.

In the current study, we confirmed the therapeutic potential of CEA-specific CAR-T cells overexpressing Bcl-xL in the treatment of colorectal cancer. Bcl-xL, a critical regulator for T cell survival, showed the potential ability to increase the persistence of genetically engineered CAR-T cells in the tumor tissues, regressed tumor growth, and increased the overall survival rate of animals. Our strategy of the CAR design may be explored as a new platform to improve CAR-T cell-based immunotherapy.

## Data Availability Statement

The raw data supporting the conclusions of this article will be made available by the authors, without undue reservation.

## Ethics Statement

The animal study was reviewed and approved by The Texas A&M University Animal Care Committee (IACUC; #2018-0006).

## Author Contributions

Conceptualization of the study was by JS. Funding acquisition, methodology, validation, supervision, and manuscript review and editing were done by HC and JS. Data curation, formal analysis, and original draft writing were done by JF, JKD, and XX. Investigation was carried out by JF, JKD, and XX. All authors contributed to the article and approved the submitted version.

## Conflict of Interest

The authors declare that the research was conducted in the absence of any commercial or financial relationships that could be construed as a potential conflict of interest.

## Abbreviations

ACT, adoptive cell transfer; AICD, activation-induced cell death; Bcl-xL, B-cell lymphoma-extra large; BSA, bovine serum albumin; CAR, chimeric antigen receptor; CEA, carcinoembryonic antigen; DMEM, Dulbecco modiﬁed Eagle medium; ICI, immune checkpoint inhibitor; IRES, internal ribosome entry site; PBS, phosphate-buffered saline; PD-1, programmed cell death-1; RT, room temperature; TNFR, tumor necrosis factor receptor.
