# Supplementary material for: Development of CAR-T Cell Persistence in Adoptive Immunotherapy of Solid Tumors
Source: Front Oncol. 2021 Jan 6;10:574860. doi: 10.3389/fonc.2020.574860 (PMC7815927; doi:10.3389/fonc.2020.574860)

Supplementary Data 1

CEA CAR

5’ - GAA CAA AAA CTC ATC TCA GAA GAG GAT CTG ATG AGC TGG GTG AAA CAG GCT CCA GGA
AAG GGT TTA AAG TGG ATG GGC TGG ATA AAC ACC TAC TCT GGA GTG CCA ACA TAT GCT
GAT GAC TTC AAG GGA CGG TTT GCC TTC TCT TTG GAA ACC TCT GTC AGC ACT GCC TAT
TTG CAG ATC AAC AAC CTC AAA AAT GAG GAC ACG TCA ACA TAT TTC TGT GCA AGA TAT GCC
TTC GGC TCT TGG TAC TTC GAT GTC AGG GGC CAA GGC ACC ACG GTC ACC GTC TCC TCA
GGT GGA GGC GGT TCA GGC GGA GGT GGC TCT GGC GGT GGC GGA TCG GAC ATC GAG
CTC ACT CAG TCT CCA GCT TCT TTG GCT GTG TCT CTA GGG CAG AGG GCC ACC ATC TCC
TGC AGA GCC AGC GAA AGT GTT GAT ACT TAT GCC GTT AGT TTT ATG AAC TGG TTC CAA
CAG AAA CCA GGA CAG CCA CCC AAA CTC CTC ATC TAT ACT GCA TCC AAG CAA GGG TCC
GGG GTC CCT GCC AGG TTT AGT GGC AGT GGG TCT GGG ACA GAC TTC AGC CTC AAC ATC
CAT CCT ATG GAG GAG GAT GAT GCT GCA ATG TAT TTC TGT CAA CAA AGT AAG GAG GTT
CCG TGG ACG TTC GGT GGA GGG ACC AAG CTG GAA ATA AAA CGG GCG GCC GCA GGT
GCG CCG GTG CCG TAT CCG GAT CCG CTG GAA CCG CGT GCC GCA TAG TTT TGG GTT
TTA GTT TGG GGA GGA GTT TTA GCT TGC TAT AGT TTA TTA GTT ACT GTT GCT TTT ATT ATT
TTT TGG GTT AGG AGC AAG AGG AGC AGG CTG CTG CAC AGC GAC TAC ATG AAC ATG
ACC CCC AGG AGG CCC GGC CCC ACC AGG AAG CAC TAC CAG CCC TAC GCC CCC CCC
AGG GAC TTC GCC GCC TAC AGG GTG AAG TTC AGC AGG AGC GCC GAC GCC CCC GCC
TAC CAG CAG GGC CAG AAC CAG CTG TAC AAC GAG CTG AAC CTG GGC AGG AGG GAG
GAG TAC GAC GTG CTG GAC AAG AGG AGG GGC AGG GAC CCC GAG ATG GGC GGC AAG
CCC CAG AGG AGG AAG AAC CCC CAG GAG GGC CTG TAC AAC GAG CTG CAG AAG GAC
AAG ATG GCC GAG GCC TAC AGC GAG ATC GGC ATG AAG GGC GAG AGG AGG AGG GGC
AAG GGC CAC GAC GGC CTG TAC CAG GGC CTG AGC ACC GCC ACC AAG GAC ACC TAC
GAC GCC CTG CAC ATG CAG GCC CTG CCC CCC AGG - 3’

CEA CAR-2A-Bcl-xL

5’ - GAA CAA AAA CTC ATC TCA GAA GAG GAT CTG ATG AGC TGG GTG AAA CAG GCT CCA GGA
AAG GGT TTA AAG TGG ATG GGC TGG ATA AAC ACC TAC TCT GGA GTG CCA ACA TAT GCT
GAT GAC TTC AAG GGA CGG TTT GCC TTC TCT TTG GAA ACC TCT GTC AGC ACT GCC TAT
TTG CAG ATC AAC AAC CTC AAA AAT GAG GAC ACG TCA ACA TAT TTC TGT GCA AGA TAT GCC
TTC GGC TCT TGG TAC TTC GAT GTC AGG GGC CAA GGC ACC ACG GTC ACC GTC TCC TCA
GGT GGA GGC GGT TCA GGC GGA GGT GGC TCT GGC GGT GGC GGA TCG GAC ATC GAG
CTC ACT CAG TCT CCA GCT TCT TTG GCT GTG TCT CTA GGG CAG AGG GCC ACC ATC TCC
TGC AGA GCC AGC GAA AGT GTT GAT ACT TAT GCC GTT AGT TTT ATG AAC TGG TTC CAA
CAG AAA CCA GGA CAG CCA CCC AAA CTC CTC ATC TAT ACT GCA TCC AAG CAA GGG TCC
GGG GTC CCT GCC AGG TTT AGT GGC AGT GGG TCT GGG ACA GAC TTC AGC CTC AAC ATC
CAT CCT ATG GAG GAG GAT GAT GCT GCA ATG TAT TTC TGT CAA CAA AGT AAG GAG GTT
CCG TGG ACG TTC GGT GGA GGG ACC AAG CTG GAA ATA AAA CGG GCG GCC GCA GGT
GCG CCG GTG CCG TAT CCG GAT CCG CTG GAA CCG CGT GCC GCA TAG TTT TGG GTT
TTA GTT TGG GGA GGA GTT TTA GCT TGC TAT AGT TTA TTA GTT ACT GTT GCT TTT ATT ATT
TTT TGG GTT AGG AGC AAG AGG AGC AGG CTG CTG CAC AGC GAC TAC ATG AAC ATG
ACC CCC AGG AGG CCC GGC CCC ACC AGG AAG CAC TAC CAG CCC TAC GCC CCC CCC
AGG GAC TTC GCC GCC TAC AGG GTG AAG TTC AGC AGG AGC GCC GAC GCC CCC GCC
TAC CAG CAG GGC CAG AAC CAG CTG TAC AAC GAG CTG AAC CTG GGC AGG AGG GAG
GAG TAC GAC GTG CTG GAC AAG AGG AGG GGC AGG GAC CCC GAG ATG GGC GGC AAG
CCC CAG AGG AGG AAG AAC CCC CAG GAG GGC CTG TAC AAC GAG CTG CAG AAG GAC
AAG ATG GCC GAG GCC TAC AGC GAG ATC GGC ATG AAG GGC GAG AGG AGG AGG GGC
AAG GGC CAC GAC GGC CTG TAC CAG GGC CTG AGC ACC GCC ACC AAG GAC ACC TAC
GAC GCC CTG CAC ATG CAG GCC CTG CCC CCC AGG TGG GCC AGG ATT CTC CTC GAC
GTC ACC GCA TGT TAG CAG ACT TCC TCT GCC CTC TCC ACT GCC ATG TCT CAG AGC AAC
CGG GAG CTG GTG GTT GAC TTT CTC TCC TAC AAG CTT TCC CAG AAA GGA TAC AGC TGG
AGT CAG TTT AGT GAT GTG GAA GAG AAC AGG ACT GAG GCC CCA GAA GGG ACT GAA TCG
GAG ATG GAG ACC CCC AGT GCC ATC AAT GGC AAC CCA TCC TGG CAC CTG GCA GAC AGC
CCC GCG GTG AAT GGA GCC ACT GCG CAC AGC AGC AGT TTG GAT GCC CGG GAG GTG ATC
CCC ATG GCA GCA GTA AAG CAA GCG CTG AGG GAG GCA GGC GAC GAG TTT GAA CTG CGG
TAC CGG CGG GCA TTC AGT GAC CTG ACA TCC CAG CTC CAC ATC ACC CCA GGG ACA GCA
TAT CAG AGC TTT GAA CAG GTA GTG AAT GAA CTC TTC CGG GAT GGG GTA AAC TGG GGT
CGC ATT GTG GCC TTT TTC TCC TTC GGC GGG GCA CGT GCG TGG AAA GCG TAG ACA AGG
AGA TGC AGG TAT TGG TGA GTC GGA TCG CAG CTT GGA TGG CCA CTT ACC TGA ATG ACC
ACC TAG AGC CTT GGA TCC AGG AGA ACG GCG GCT GGG ATA CTT TTG TGG AAC TCT ATG
GGA ACA ATG CAG CAG CCG AGA GCC GAA AGG GCC AGG AAC GC TTC AAC CGC TGG TTC
CTG ACG GGC ATG ACT GTG GCC GGC GTG GTT CTG CTG GGC TCA CTC TTC AGT CGG AAA
TGA - 3’

Supplementary Data 2 - 1

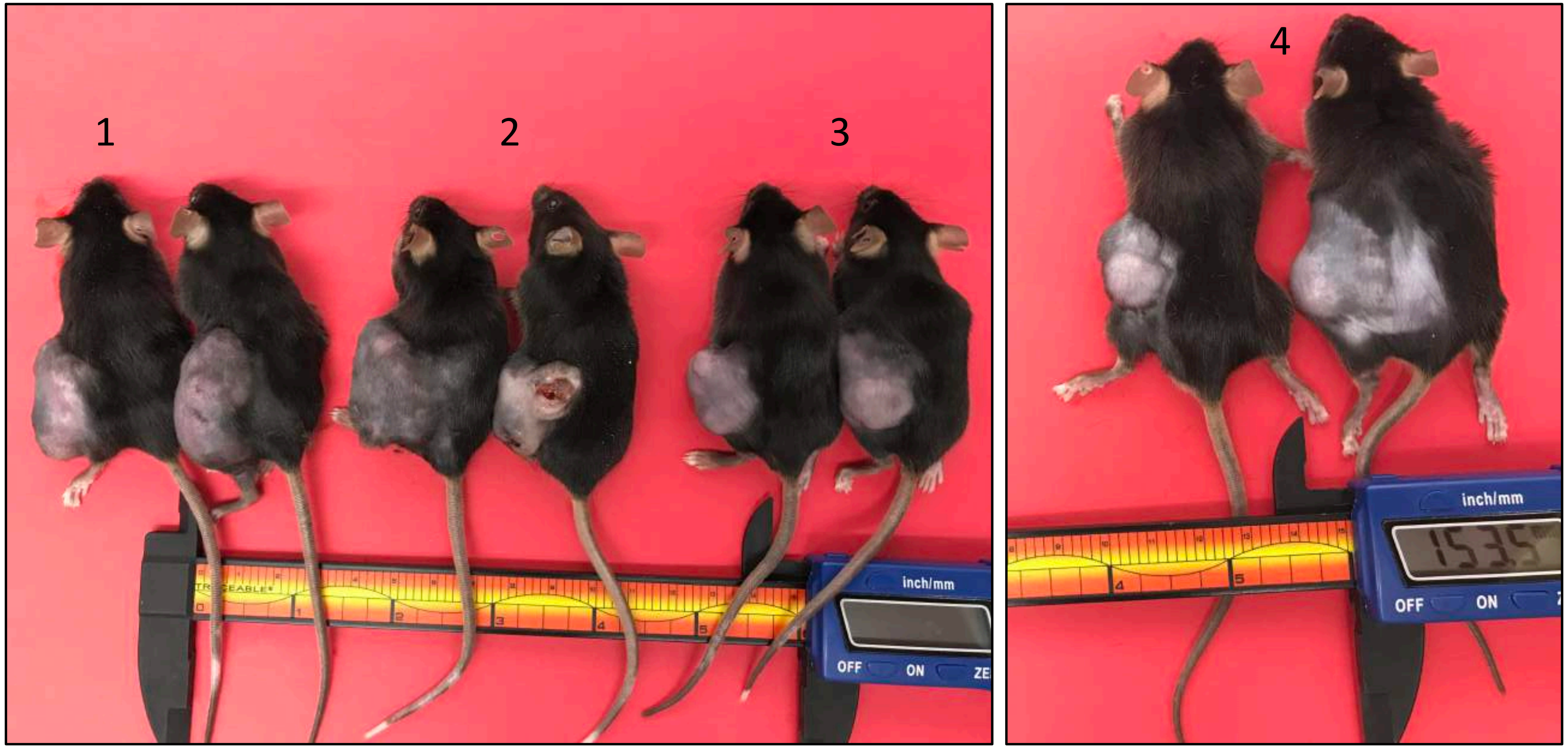

On mice

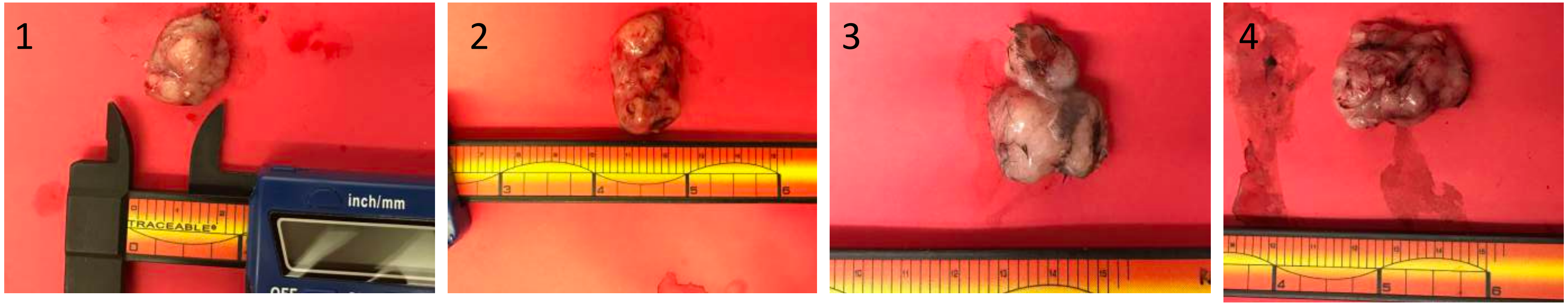

Explanted

Supplementary Data 2 - 2

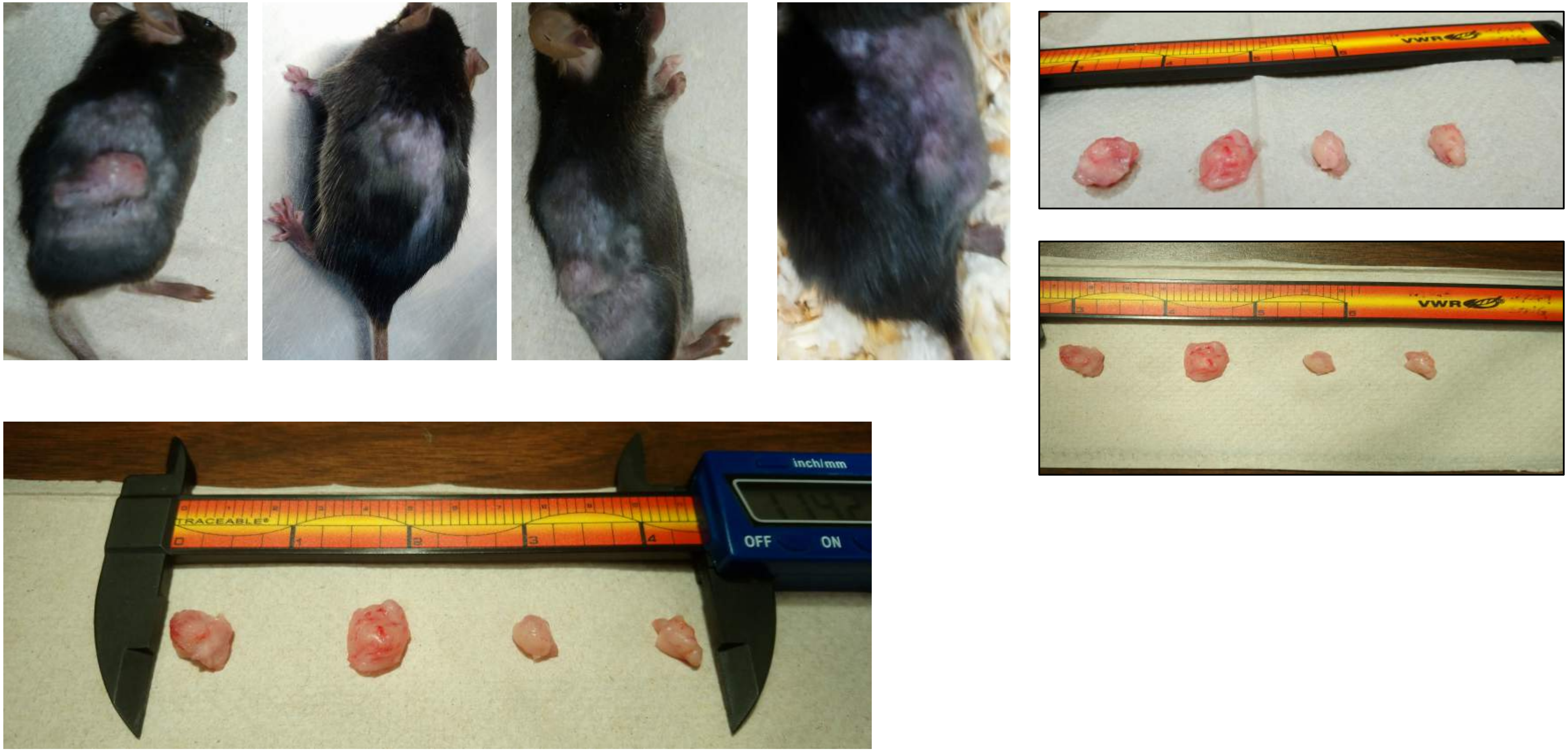

Supplement: Supplementary file 1 [file DataSheet_1.pdf]
